# Influence of Molecular Weight on Thermal and Mechanical Properties of Carbon-Fiber-Reinforced Plastics Based on Thermoplastic Partially Crystalline Polyimide

**DOI:** 10.3390/polym15132922

**Published:** 2023-07-01

**Authors:** Gleb Vaganov, Maria Simonova, Margarita Romasheva, Andrey Didenko, Elena Popova, Elena Ivan’kova, Almaz Kamalov, Vladimir Elokhovskiy, Vyacheslav Vaganov, Alexander Filippov, Vladimir Yudin

**Affiliations:** 1 Institute of Macromolecular Compounds of Russian Academy of Sciences, Bol’shoy pr. 31, St. Petersburg 199004, Russia; mariasimonova1983@mail.ru (M.S.); rita.romasheva@gmail.com (M.R.); vanilin72@yandex.ru (A.D.); men682003@mail.ru (E.P.); ivelen@mail.ru (E.I.); spb.kamalov@gmail.com (A.K.); vladimir.elokhovskiy@gmail.com (V.E.); afil@imc.macro.ru (A.F.); yudinve@gmail.com (V.Y.); 2 Higher School of Automation and Robotics, Peter the Great St Petersburg Polytechnic University, Polytechnicheskaya St. 29, St. Petersburg 195251, Russia; prvaganov_spb@mail.ru

**Keywords:** carbon-fiber-reinforced plastic, molecular weight, thermal and mechanical properties, interlayer fracture toughness

## Abstract

For the first time, a study of the influence of the molecular weight of the thermoplastic partially crystalline polyimide R-BAPB on the thermophysical and mechanical properties of carbon plastics was presented. The molecular weight of polyimide was determined using the method of light scattering and the study of the intrinsic viscosity of polyamic acid solutions. To obtain CFRPs, the uniform distribution of polyimide powder on continuous carbon fibers via electrostatic spraying and further hot calendering and pressing were applied. The study of the structure of the obtained carbon plastics via scanning electron microscopy has shown that the growth of the molecular weight of polyimide prevents the impregnation of carbon fiber with the introduced polyimide. Moreover, an increase in the molecular weight of polyimide leads to a rise in glass transition and thermal decomposition temperatures up to 590 °C, while the degree of crystallinity of CFRP falls. Nonetheless, raising the molecular weight from 22,000 to 70,000 g/mol of a binder polymer improves the interlayer fracture toughness G_1C_ by more than five times.

## 1. Introduction

At present, much attention is paid to receiving new composite materials based on thermoplastics reinforced with continuous fibers. Relative to the traditional composite materials based on thermosetting binders, composite materials based on thermoplastic polymers have the following advantages: firstly, thermoplastics have high impact strength, good resistance to certain chemicals, and low moisture absorption compared to thermosets; secondly, the short cycle times, made possible with thermoplastics, make them a good potential alternative due to the significant reduction in manufacturing costs compared to thermoset binders [1,2]. An additional advantage of using thermoplastics as a matrix for obtaining carbon fiber is their unlimited shelf life [3].

The use of high-tech thermoplastics in combination with continuous carbon fibers is of particular interest in the development of fibrous composite materials. In addition to the traditional advantages of thermoplastic polymers (suitability for reuse; resistance to chemicals, solvents, and radiation; unlimited shelf life; the possibility of repairing, remelting, and processing; etc.), they usually have improved mechanical properties such as high level of impact strength, strength, stiffness, and fatigue resistance [2,4]. Moreover, they retain their mechanical characteristics at high temperatures and show thermal stability even during long-term use [5].

To date, polysulfones [6], polyether ether ketones [7], polyphenylene sulfides [6], polyphenylene oxides [8], and polyimides (PI) [9,10] are studied as promising thermoplastics for the production of carbon-fiber-reinforced plastics (CFRPs) due to their excellent mechanical properties, heat, and chemical resistance. Among this group of thermoplastics, the most interesting binders to produce heat-resistant carbon fiber plastics are semicrystalline thermoplastic polymers [11,12]. The development of high-tech semicrystalline thermoplastics can serve as an effective way to improve the thermal stability, resistance to solvents, and mechanical properties of composites based on them [11,13]. One of the brightest representatives of highly heat-resistant thermoplastic binders capable of recrystallization from a melt is polyimide R-BAPB based on the dianhydride R (1,3-bis(3,3′,4,4′-dicarboxyphenoxy)benzene) and the diamine BAPB (4,4′-bis(4″-aminophenoxy)biphenyl) [14]. Polyimide R-BAPB has a high glass transition temperature of about 200 °C and a viscosity of about 1000 Pa*s at 360 °C. A feature of this thermoplastic is the ability to control crystallization and recrystallization, which has been repeatedly demonstrated [15,16] in the example of fibers and binders for composite materials. The operating temperature of CFRPs with R-BAPB matrix in the crystallized state can reach 300 °C. 

In the development of CFRPs based on high-tech thermoplastics, an important characteristic of the polymer is its molecular weight. Thermoplastics used qua binders of the carbon fibers have a disadvantage associated with lower impregnation of reinforcing fibers compared to thermosetting binders. The reason is the high melt viscosity of the thermoplastic resin. On the one hand, the molecular weight will affect the viscosity of the thermoplastic binder for the composite material and, on the other hand, affect the mechanical properties of CFRP. The authors of [17] studied the influence of the molecular weight of the thermoplastic polyester-cardo resin (PEK-s) on the thermal, mechanical, and interfacial properties of the composite. The research results showed that composites with a higher molecular weight of the polymer have higher mechanical properties due to a higher level of interfacial adhesion. In [18], it was found that for the original polyethersulfone, the higher the molecular weight, the better both mechanical and tribological characteristics. However, some opposite trends were observed for composites as follows: the lower the molecular weight, the higher the mechanical properties of the composite. The authors of [19] showed that by increasing the molecular weight of polyethersulfone (from 14,000 to 28,000 g/mol), it was possible to increase the values of the interlaminar fracture toughness of CFRP by about 46%. At the same time, up to now, only a few works about the effect of the molecular weight of high-temperature thermoplastics on the properties of carbon-fiber-reinforced plastics based on them have been published. Thus, achieving high mechanical characteristics requires careful selection of the optimal molecular weight of the thermoplastic polymer under study. Until now, such detailed studies have not been carried out on the class of heat-resistant crystallizing polyimides. Therefore, this present work is devoted to estimating the molecular weight of the synthesized thermoplastic polyimides R-BAPB and studying the effect of changing the molecular weight on the properties of carbon plastics.

## 2. Experimental

### 2.1. Materials

1,3-bis(3′,4-dicarboxyphenoxy)benzene (dianhydride R) with Tm ~164 °C was obtained from TechKhimProm LLC, Yaroslavl, Russia. 4,4′-bis(4″-aminophenoxy)biphenyl) (diamine BAPB) with Tm ~198–199 °C was obtained from VWR International. Phthalic anhydride (PA) was used as a chain limiter for the polycondensation reaction, and Tm ~131–134 °C was obtained from Sigma-Aldrich Co. LLC, St. Louis, MO, USA. Acetic anhydride, benzene, and triethylamine were supplied by Sigma-Aldrich Co. LLC.

To obtain CFRPs, continuous carbon fibers of the ELUR P-0.08 type (Russia) were used as a reinforcing filler in the form of a tape 200 mm wide with a linear density of 15 g.

### 2.2. Synthesis of Polymers

The synthesis was carried out using the method of chemical imidization in accordance with the protocol described in [15]. In the first stage, the polycondensation of anhydride with diamine to prepolymer polyamic acid (PAA) occurred in a solution of dimethylacetamide (DMAA) solvent at a temperature of 15 °C. The molecular weight of each obtained prepolymer was controlled by violating the stoichiometric ratio of the initial monomers during the formation of polyamic acid. In this case, the closer the ratio of mole fractions of dianhydride to diamine, the higher the molecular weight. This ratio was changed from 0.99 to 0.93. The chain growth process was interrupted by introducing PA into the reaction mixture for chemical deactivation of the end groups of the macromolecule. Next, chemical imidization was carried out at the temperature of 30 °C. Chemical imidization consisted of the cyclization of the polyamic acid units of the PAA solution in DMAA via the chemical agents of the imidizing mixture. The calculation of the imidizing mixture was carried out based on the molar ratio of amido acid units in the elementary unit of polyamido acid; triethylamine and acetic anhydride were taken in a threefold excess (by moles), and the number of moles of benzene was taken in one and a half excess. The resulting powdery precipitate was filtered through a Schott filter and washed with sulfuric ether and methyl alcohol to remove residual solvent and imidizing mixtures. The resulting powders were subjected to vacuum drying for 2 h at the temperature of 220 °C. As a result of the synthesis, semicrystalline powders of R-BAPB were obtained with different ratios of dianhydride to diamine: 0.93, 0.95, 0.97, 0.98, and 0.99.

### 2.3. Preparation of Carbon-Fiber-Reinforced Plastics Based on Polyimide R-BAPB with Different Molecular Weights

Based on the synthesized polyimide powders, unidirectional carbon plastics were obtained via the formation of a prepreg using a technological and environmentally friendly approach based on the uniform distribution of polyimide powder on a carbon tape using electrostatic spraying followed by their impregnation in heated calenders and hot pressing. The peculiarity of this impregnation method is that due to the electrostatic spraying of the powder over the surface of the reinforcing fibers, the distance over which the polymer melt must spread is reduced. In this regard, it is possible to obtain a prepreg with good impregnation even when using high-viscosity thermoplastic binders [20,21,22]. 

The process to obtain samples of CFRPs based on polyimide R-BAPB consisted of several operations, similar to those described in [23]. 

The resulting synthesis of the polyimide powder with different molecular weights was crushed and sifted through a 125 µm sieve. As a result, the powder particles were formed with a particle size not exceeding 125 microns. Blanks, 30 × 15 cm in size, were cut from a carbon tape for the subsequent introduction of polyimide powder into them by an electrostatic sprayer. A carbon tape coated with polyimide powder was placed between the sheets of the polyimide film and rolled through the calenders heated up to 360 °C. In the process of heat treatment, the fibers are moistened by molten polyimide, which spreads and penetrates into the interfiber space under the action of capillary forces. The prepregs obtained as a result of rolling through the calenders were cut into blanks with dimensions of 15 × 3.2 cm. 

The blanks were stacked and packed in 36 layers in a mold with 0° orientation between the layers. From one edge in the middle (between the 18th and 19th layers), a polyimide film 3 cm long and 3.2 cm wide was laid to obtain a crack in this place, which was necessary for the subsequent testing of CFRP for interlayer fracture toughness. Then, the prepregs were pressed at a pressure of 1 MPa. 

Pressing was carried out according to the following regime: heating from room temperature up to 360 °C at a heating rate of 5 °C/min; exposure at the temperature of 360 °C for 10 min; cooling from 360 °C to 280 °C; exposure at the temperature of 280 °C for 1 h; cooling from 280 °C to room temperature under pressure. For all CFRPs, the fiber content varied within 64–70 vol.%. To study the bending strength, plates with plates ~1 mm thick and ~4 mm wide were cut from the obtained carbon plastics.

### 2.4. Measurements

The molecular weight of the synthesized polyimides was calculated on the basis of the study of the PAA prepolymer since the R-BAPB polyimide is an insoluble polymer.

The absolute molar masses (MM) and the hydrodynamic radii *R*_h-D_ of macromolecules are determined using static (SLS) and dynamic light scattering methods [24] (DLS) in dilute solutions in dimetil acetamid DMAA (density ρ_0_ = 0.93 g∙cm^−3^, dynamic viscosity η_0_ = 1.02 cP, and refractive index *n*_0_ = 1.4356). The experiments were performed using Photocor Complex instrument (Photocor Instruments Inc., Moscow, Russia), which is equipped with a Photocor DL a diode laser (wavelength *λ* = 632.8 nm and power 5–30 mW). For investigated solutions in DMAA, the distribution of the light scattering intensity *I* over the hydrodynamic radii *R*_h-D_(*c*) of scattering objects was unimodal. The values of *R*_h-D_(*c*) were determined in the wide concentration range and extrapolated to zero concentration to obtain the hydrodynamic radius *R*_h-D_ of macromolecules [25].

As is well known, the translation diffusion coefficients *D*_0_ and the friction coefficient *f* of macromolecules are related to *R*_h-D_ via Stokes–Einstein equations as follows [26]:

*D*_0_ = *k*_B_*T/f = k*_B_*T/6*πη_0_*R*_h-D_ (1)

where *k*_B_ is Boltzmann’s constant, and *T* is the absolute temperature.

SLS measurements were performed at the angle of 90° since no angular dependence of the scattered light was observed. The obtained results were analyzed according to the Debye method, and the values of the weight-average molar masses *M_w_* and the second virial coefficient *A*_2_ were calculated using the following formula [26]:

(2) $$\frac{cH}{I_{90}}=\frac{1}{M_{w}}+2A_{2}c$$

where *H* is the optical constant [26].

(3) $$H=\frac{4\pi^{2}n_{{}_{0}}^{2}{\left(dn/dc\right)}^{2}}{N_{A}\lambda_{0}^{4}}$$

Here, *I*_90_ is the excessive intensity of light scattered at an angle of 90 °, *N*_A_ is Avogadro’s number, and *dn*/*dc* is the refractive index increment. The values of *dn*/*dc* were determined using an RA-620 refractometer (Shimadzu, Kyoto, Japan) with a wavelength *λ*_0_ = 589.3 nm. The values of *dn*/*dc* were calculated from the slope of concentration dependences of the difference Δ*n* = *n* − *n*_0_ between refractive indexes of the solution *n* and the solvent *n*_0_. 

The viscometry experiments were performed using an Ostwald-type capillary viscometer (Cannon Instrument Company Inc., State College, PA, USA). The dependencies of the reduced viscosity η_sp_/*c* on the concentration were analyzed using the Huggins equation as follows [27]:

η_sp_/*c* = [η] + *k*_H_[η]^2^*c* (4)

where [η] is the intrinsic viscosity, and *k*_H_ is the Huggins constant.

Light scattering, viscometry, and refractometry experiments were carried out at 21 °C. Millipore filters (Millipore Corp., Billerica, MA, USA) with a PTFE membrane with a pore size of 0.20 nm were used. Light scattering, viscometry, and refractometry experiments were performed at 21 °C. 

To confirm the chemical structure of the PI powder formed after the chemical imidization of PAA, an IRA-Affinity-1S IR-Fourier spectrometer (single-beam Michelson-type interferometer with an incidence angle of 30°) was used. The spectral range was 7800–350 cm^−1^, the signal-to-noise ratio was >30,000:1, and the maximum resolution was 0.5 cm^−1^.

The viscosity of R-BAPB polyimide melts was determined on an MCR-301 rheometric system from Anton Paar (Austria) using a cone–plane pair. The test was carried out in an oscillating mode in the frequency range from 100 rad/s to 1 rad/s at a temperature of 360 °C, corresponding to the temperature of further production of carbon fiber during calendering and hot pressing.

The structure of the CFRPs based on R-BAPB polyimide was studied using scanning electron microscopy. Micrographs of the fracture surface of the carbon plastics were obtained using a scanning electron microscope (SEM) Supra-55 VP (Carl Zeiss, Germany) using a secondary electron detector. To obtain a high-quality fracture surface, the sample was destroyed in liquid nitrogen. The resulting transverse cleavages of the samples were fixed with a special conductive adhesive on microscope holders and a thin layer of platinum was applied. The accelerating voltage was 5 kV. In addition, using SEM, morphology of the fracture surface of CFRPs was analyzed after testing the interlayer fracture toughness. 

Thermal properties of the samples were researched via differential scanning calorimetry (DSC) on a NETZSCH instrument (Germany) DSC 204 in accordance with ISO 11357-3:2011. The tests were carried out in the temperature range from 30 to 400 °C at a heating rate of 10 °C/min in an inert atmosphere (argon). The DSC curves were used to determine the glass transition and melting temperatures, as well as the enthalpy of melting of the crystalline phase. 

To calculate the degree of crystallinity of CFRPs, there was used the enthalpy of melting of 100% crystal (ΔH0) determined for R-BAPB in the alike earlier work, which was found to be 90 J/g [14]. Determination of the degree of crystallinity was carried out according to the formula χ = ΔHm/ΔH0, where ΔHm is the melting enthalpy of the polymer, determined during the DSC experiment. To calculate the enthalpy of melting of the polymer, mass of the CFRP sample minus the mass of the carbon fibers was taken as given data. 

To determine the beginning of the process of thermal decomposition in carbon plastics, the method of thermogravimetric analysis (TGA) was applied using a TG 209 F1 instrument (NETZSCH, Germany) according to ASTM E1877-21. The tests were carried out at temperatures from 30 to 700 °C at a heating rate of 10 °C/min in an inert atmosphere (argon).

The temperature dependences of the loss modulus (E′) were determined using mechanical analysis (DMA) on a DMA 242 C setup (NETZSCH, Germany) in the three-point bending mode according to ASTM D4065. The measurements were carried out at a frequency of 1 Hz, a deformation amplitude of 0.1%, and a temperature rise rate of 5 °C/min. ASTM D7028-07(2015).

Interlayer fracture toughness G_1C_ (crack resistance) was studied on CFRP samples with the following characteristic dimensions: width of 15 mm, length of 120 mm, thickness of 3 mm, and initial crack length of 30 mm. The carbon fiber samples were tested on a tensile testing machine 1958U-10-1 (Russia) at room temperature using the “double cantilever beam” (DCB) method in accordance with ASTM D5528/D5528M-21. The loading rate along the crack edges was 10 mm/min. A more detailed description of the definition of conducting an experiment to determine the interlayer fracture toughness is described in a similar earlier work [23]. The quantity of G_IC_ was averaged over five measurements during propagation of the crack for each of CFRP samples.

Bending strength tests (σb) of CFRP were carried out via the three-point bending method on the same tensile testing machine at room temperature. Three-point bending test specimens were plates ~1 mm thick and ~4 mm wide. The distance between the supports was 30 mm, and the sample loading rate was 2 mm/min. The bending strength and modulus of the CFRPs were determined via statistical averaging of the measurements for at least five samples.

## 3. Results and Discussion

### 3.1. Polymer Synthesis

**Figure 1:**
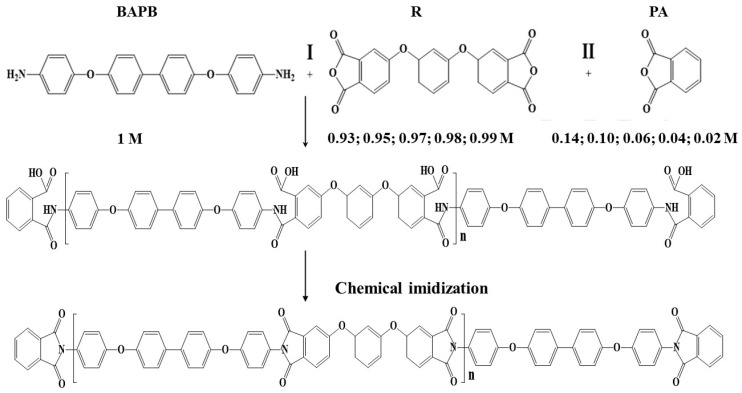
Scheme for obtaining polyimide R-BAPB of different molecular weights.

As a result of the synthesis, PAA and powders of partially crystalline PI R-BAPB with different molar ratios of dianhydride to diamine were obtained as follows: 0.93, 0.95, 0.97, 0.98, and 0.99. The designation of the obtained PAA and the PI obtained on their basis are presented in Table 1. The scheme for obtaining polyimide is shown in Figure 1. 

The study of the chemical structure of the homologous series of polyimide via IR-Fourier spectroscopy demonstrates typical bands for the imide cycle as follows: at 1780 cm^−1^ (C=O asymmetric stretching), at 1715–1720 cm^−1^ (C=O symmetric stretching), and at 735 cm^−1^ (C=O) with a peak of C-N stretching vibrations at 1370 cm^−1^. The spectrum of polyimide for the ratio of the mole fractions of dianhydride to diamine 0.95 is given in the previous paper [15]. As a result, the FT-IR spectra confirmed that PI was completely imidized. 

### 3.2. Structure Conformational and Molecular Hydrodynamic Characteristics

As mentioned above, the molecular weight of polyimide was calculated based on the measurement of the molecular weight of the prepolymer PAA.

The values *M_w_* and *R*_h-D_ are presented in Table 2. Table 2 shows that using DMAA makes it possible to obtain molecular dispersion solutions of PAAs. The virial coefficients have high positive values and decrease with increasing MM of polymers. With the increase in the content of dianhydride R from 0.93 to 0.99 in the reaction mixture, the values of molar masse, intrinsic viscosity [η], the hydrodynamic radius *R*_h–D_ of macromolecules, and the translational friction coefficient *f*, grow.

Using the *M_w_* values, it is easy to calculate the polymerization degree *N* of investigated samples (the molar masse of repeating units is close to 734 g∙mol^−1^). The *N* values are listed in Table 2. Table 2 also presents the average values of the contour length *L* of the polymer chain. Length *L* was calculated using length of monomer units *λ*_0_ = 3.25 nm (HyperChem program 6.03). 

For the primary analysis of the hydrodynamic and conformational polymers, it is convenient to use scaling relations of the Kuhn−Mark−Houwink−Sakurada (KMHS) type for intrinsic viscosity [27]

[η] = *K*_η_*M^a^* (5)

and translation frictional coefficient [27]

*f* = *K*_f_*M^b^* (6)

**Figure 2:**
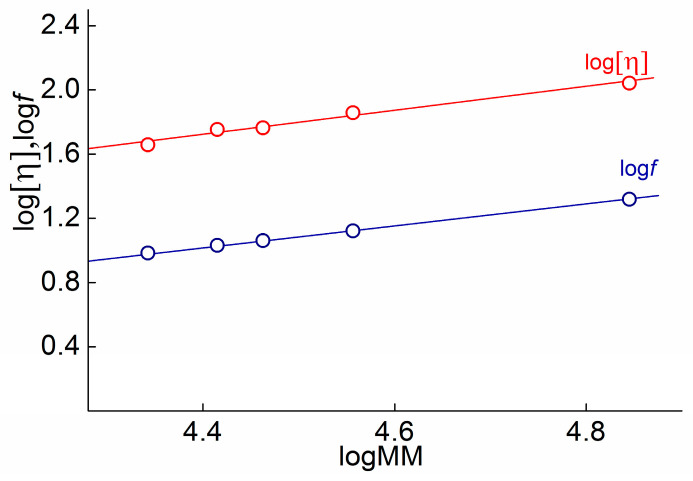
KMHS dependences of intrinsic viscosity [η] and frictional coefficient *f* for PAAs in DMAA in solutions at 21 °C.

Exponents *a* and *b* of the KMHS equations are sensitive indicators of the macromolecular shape. Figure 2 shows the KMHS plots obtained for the investigated polymer. One can see that for both hydrodynamic characteristics, the experimental points correspond to straight-line KMHS dependencies. The obtained values of *a* = 0.83 ± 0.04 and *b =* 0.66 ± 0.06 are typical for flexible chain polymer in thermodynamic solvent. 

The values of the hydrodynamic invariant *A*_0 are_ also evidence of the increased flexibility of the PAAs chains. This characteristic is determined by the experimental values of molar mass *M*, intrinsic viscosity [η], and translation diffusion coefficient *D*_0_ by the formula [28].

(7) $$A_{0}=\eta_{0}D_{0}{\left(\frac{M\left[\eta \right]}{100}\right)}^{1/3}/T$$

It is clearly seen that the *A*_0_ values hydrodynamic invariant for PAAs samples do not change with increasing MM, and the average value *A*_0_ = (3.1 ± 0.2)∙10^−10^ erg·K^−1^·mol^−1/3^ are in good agreement with the experimental and theoretical values of *A*_0_ = 3.2∙10^−10^ erg·K^−1^·mol^−1/3^ for linear flexible-chain polymers [29]. Note that the value of *A*_0_ is constant in wide intervals of polymer molar mass and depends on molecular conformational and architecture. In particular, for flexible chain polymers and rigid chain polymers, the average experimental values of hydrodynamic invariant differ by almost 20%: *A*_0_ = 3.2∙10^−10^ erg·K^−1^·mol^−1/3^ [30] and 3.8∙10^−10^ erg·K^−1^·mol^−1/3^, respectively. Low values of *A*_0_ ≤ 2.8, i.e., lower than the theoretically predicted value for a hard sphere, are typical for polymers with complex architecture, such as molecular brushes [31], as well as hyperbranched [32] and star-shaped polymers [33]. 

### 3.3. Equilibrium Rigidity of PAAs

The measure of equilibrium stiffness is the length *A* of the statistical segment. As mentioned above, the samples were studied in a thermodynamically good solvent. Accordingly, to determine the value of the Kuhn segment length, one should use theories that take into account volume effects, namely, theories of Stockmayer-Fixman [34] for intrinsic viscosity

[η]/*M*^1/2^ = (*LA/M*)^3/2^Φ_∞_ + 0.51Φ_∞_*BM*^1/2^ (8)

and Cowie-Bywater [35] for translation friction coefficient

*f*/*M*^1/2^ = η_0_*P***_∞_**(*LA/M*)^1/2^[1 + 0.2*B*(*M*/*LA*)^3/2^*M*^1/2^] (9)

where Φ_∞_ = 2.84 × 10^23^ mol^−1^ is Flory parameter, and *P*_∞_ = 5.11 is the hydrodynamic parameter. The parameter *B* depends on the thermodynamic quality of the solvent; for a *θ*–solvent, *B* = 0. Therefore, using an extrapolation procedure to zero molar mass, the unperturbed dimensions of polymer chains can be found.

**Figure 3:**
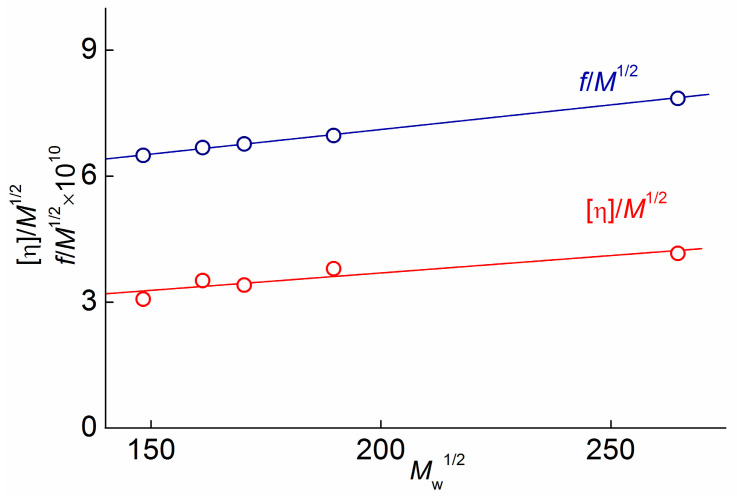
Extrapolations of Stockmayer–Fixman, [η]/*M*^1/2^ on *M*^1/2^ and Cowie-Bywater *f*/*M*^1/2^ on *M*^1/2^) for PAAs in DMAA.

Figure 3 demonstrates the dependencies of [η]/*M*^0.5^ and *f*/*M*^0.5^ on *M_w_*^1/2^ obtained for the polymer understudy. Both dependencies can be approximated by straight lines, and the Kuhn segment length *A* was derived from their intercepts using Equations (8) and (9). Using viscometry data yields a Kuhn segment length *A*_η_ = (2.2 ± 0.3) nm, and the analysis of the translational friction data yields *A*_f_ = (2.0 ± 0.2) nm. The obtained values of *A*_η_ and *A*_f_ confirm the above assumption that PAAs are a flexible-chain polymer. These values are in agreement with the magnitudes of *A* reported for polyimides of different chemical structures with swing joint groups or flexible spacers. In particular, Kuhn segment lengths for aromatic polyimides were found (experimentally and theoretically) to be 1.9–3.8 nm [36], 2.5–4.2 nm [37], 1.6–4.4 nm [38], 2–3 nm [39], and 1–2 nm [40]. The high flexibility of the investigated polymers is caused by multiple internal rotation degrees within the monomer unit of the PAAs around Ph-O bonds. It should be noted that the Kuhn segment A increases to 2.6 nm after the cyclization of the studied amido acids and transformation into polyimide, which is shown in [41]. 

### 3.4. Investigation of the Viscosity of the Polyimide Melt

**Figure 4:**
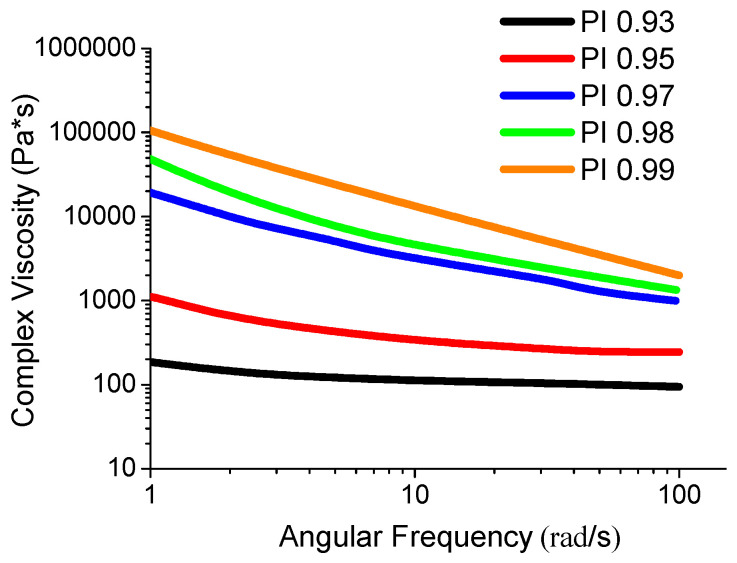
Dependence of the viscosity of melts of the R-BAPB with different molecular weigh on the angular frequency.

An important characteristic in the production of carbon-fiber-reinforced plastics based on thermoplastic binders is the value of the molten polymer’s viscosity. At a high viscosity of the melt, the impregnation of the fibers deteriorates, and, therefore, it is no longer possible to obtain defect-free carbon plastics. In this regard, the viscosity of the polyimide melt of different molecular weights was studied (Figure 4).

Angular frequency viscosity studies of R-BAPB melts as a function of molecular weight showed that as the molecular weight increases, an elevation in viscosity is observed, especially at low shear rates. The composition with a low molecular weight PI 0.93 is characterized by an almost Newtonian melt flow. As the molecular weight rises, the dependence of viscosity on frequency begins to appear (starting from sample PI 0.95). When moving to higher molecular weight R-BAPB homologs (PI 0.97, PI 0.98, and PI 0.99), the overall level of system viscosity increases, as well as the appearance of a strong effect of changes in melt viscosity on angular frequency. Thus, at the frequency of 100 rad/s, the R-BAPB melt viscosity for PI 0.97 is ≈1000 Pa*s. Lowering the angular frequency to 10 rad/s leads to rocketing melt viscosity up to ≈3000 Pa*s (see Figure 4). Thus, the structuring of the R-BAPB polyimide melt at low strain rates in the case of high-molecular homologs manifests itself. This fact is caused by the high molecular weight: the long macromolecules tend to intertwine and tangle, and thereby an increase in viscosity occurs, which is especially pronounced at low strain rates [42]. The highest meaning of the melt’s viscosity is observed for the highest molecular weight sample PI 0.99. Such a high melt viscosity for the high molecular weight homolog can hinder the high-quality impregnation of the carbon fibers with thermoplastic polyimide. 

### 3.5. Investigation of Thermal Properties of CFRP

The analysis of the obtained DSC and TGA data for the carbon plastics with different molecular weights of polyimide is represented in Table 3. With an increase in the molecular weight, regular growth in the glass transition temperature, melting temperature, and lowering degree of crystallinity of the polymer is observed. Moreover, the most acute reduction from 40% to 1% is revealed when going from sample PI 0.98 to PI 0.99, respectively (see Table 3). Such a strong decrease in the degree of crystallinity is due to the fact that with an increase in molecular weight, the mobility of longer macromolecules is hindered, which prevents the packing of the macromolecules and the formation of a crystalline phase.

Based on the obtained TGA data, it can be concluded that all the studied polyimide carbon plastics are stable up to the temperature of ≈570 °C, after which the processes of thermal destruction begin to become more active. With an increase in molecular weight, there is a slight enchantment in the thermal degradation temperature τ5 (the temperature at which a loss of 5% of the polymer mass occurs), especially when moving to the CFRP sample with the highest molecular weight.

### 3.6. Investigation of Structure of CFRP

**Figure 5:**
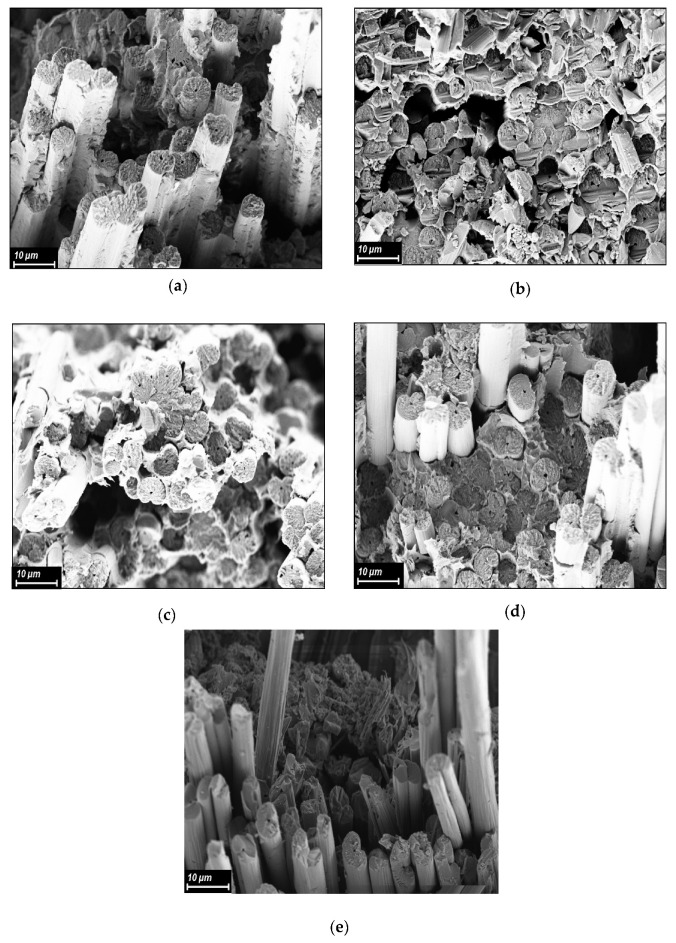
SEM images the fracture surface of the CFRPs based on the polyimide R-BAPB with different molecular weights: (**a**) PI 0.93, (**b**) PI 0.95, (**c**) PI 0.97, (**d**) PI 0.98, and (**e**) PI 0.99.

It is known that the mechanical properties of composite materials are affected by the quality of impregnation of reinforcing fibers with a binder matrix, which was studied by some researchers [43]. Impregnation with the binder matrix depends on many factors such as surface tension, capillarity, and viscosity, the latter being considered the most important. To study the impregnation of the binders based on R-BAPB of various molecular weights of the carbon fibers, transverse cleavages of unidirectional CFRPs were studied using SEM. In Figure 5, one typical image for each CFRP sample is shown. The absence of obvious voids in the CFRP samples indicates that the matrices have completely impregnated the fiber bundles. Otherwise, if only partial impregnation had occurred due to high viscosity or other reasons, the porosity would have been evident in the SEM images, as reported elsewhere [44]. The absence of the obvious voids inside the carbon-fiber-reinforced plastics based on polyimide samples PI 0.93, PI 0.95, and PI 0.97 indicates that the polyimide binders have completely impregnated the carbon fibers, which are evenly distributed over the composite CFRP sample (Figure 5a–d). In contrast, for the CFRP based on PI 0.99, there are areas where the fibers are not impregnated with polyimide binder (Figure 5e), which is probably due to the high melt viscosity of polyimide PI 0.99. 

### 3.7. Investigation of Thermomechanical Properties of CFRP

**Figure 6:**
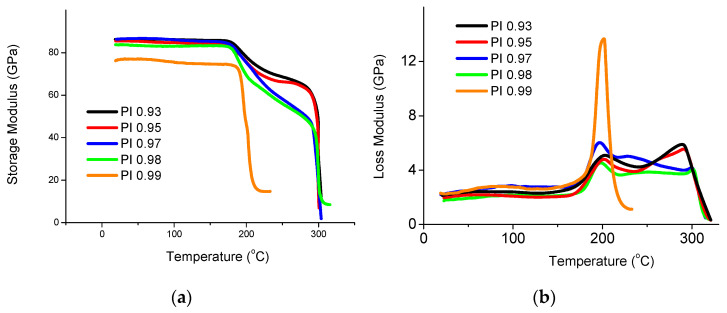
Dependences of storage modulus (**a**) and loss modulus (**b**) on the temperature recorded for the CFRPs based on R-BAPB with different molecular weights.

The results of studies of the temperature dependences of the storage moduli E′ and losses E″ of the CFRPs are shown in Figure 6. The temperature dependence curve of E″ has one narrow maximum for CFRP based on the highest molecular weight homolog PI 0.99 at the temperature of ≈201 °C. Unstiffening segments of the polyimide macromolecules cause the presence of this peak. When moving to the carbon plastics based on lower molecular weight polyimide samples, the E″ maxima become wider, and even distinct additional maxima appear in the region of higher temperatures of about 300 °C, which is clearly observed for carbon plastics based on PI 0.95 and PI 0.93 (Figure 6b). The change in the nature of the curves of the dependence of the loss modulus on temperature is due to the presence of the crystalline phase in the polymer (see Table 2). 

After considering the temperature dependence of the storage modulus (E′) for all carbon plastics of the R-BAPB homologous series, except polyimide with the ratio of the matrix component equal to 0.99, it can be stated the two-stage mechanism for reducing the modulus with increasing temperature is most probably observed, which is associated with the presence of the crystalline phase in the polymer (Figure 6a). For the CFRPs based on PI 0.93, PI 0.95, PI 0.97, and PI 0.98, the initial moduli of elasticity vary from 83 to 86 GPa. Initially, a decrease in the moduli manifests itself at the temperatures of the beginning of unfreezing the segmental mobility of the polymer (above 185 °C). After some drop, the moduli of elasticity remain at a high level up to the temperature of ≈300 °C. However, it should be noted that the magnitude of the drop in the moduli of elasticity depends on the molecular weight of the polyimide used. For the CFRP samples PI 0.93 and PI 0.95, the elastic moduli decrease down to ≈60 GPa. In the case of using higher molecular weight homologs of R-BAPB (samples PI 0.97 and PI 0.98), the initial decrease in the moduli is more significant down to ≈45 GPa. The difference in the rate of modulus change is associated with different degrees of crystallinity of carbon-fiber-reinforced plastics depending on the molecular weight (see Table 3). On the contrary, the highest molecular weight sample of polyimide PI 0.99 is described by a one-step drop in the modulus above the temperature of 185 °C. Such a decrease in the modulus is associated with the transition of the polymer matrix from a glassy state to the highly elastic one. It should be noted that the modulus of elasticity of the CFRP PI 0.99 at room temperature has the lowest value among all carbon fiber plastics, namely, the value of the modulus of elasticity does not exceed 75 GPa. Such a low value of the elastic modulus is probably due to the fact that, because of the high viscosity of the PI 0.99 polyimide melt, it is not possible to carry out high-quality impregnation of each carbon fiber with the polymer binder during the production of carbon plastic and, therefore, to effectively transfer the load from the polymeric matrix to the carbon fibers. Thus, owing to the presence of the crystalline phase, the CFRPs based on PI 0.93, PI 0.95, PI 0.97, and PI 0.98 can withstand loads up to temperatures of ≈300 °C. In the case of the CFRP PI 0.99, the operating temperature is limited by its glass transition temperature.

### 3.8. Investigation of Mechanical Properties of CFRP

**Figure 7:**
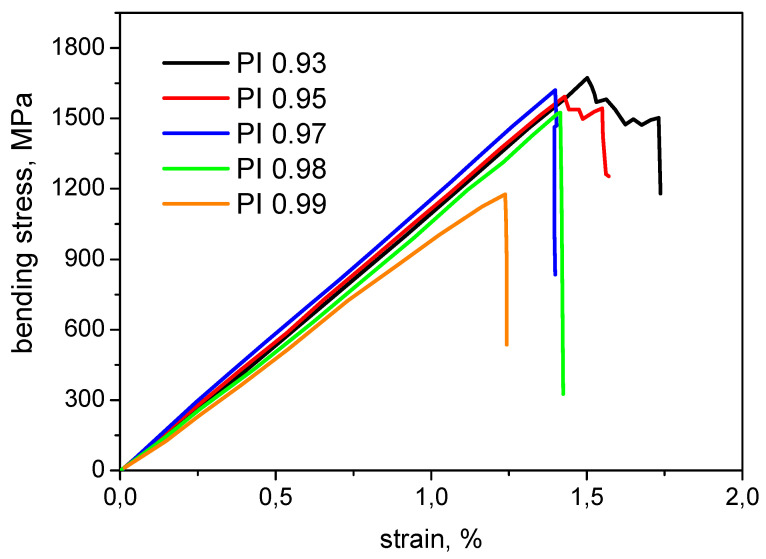
Stress–strain curves of polyimide carbon-fiber-reinforced plastics in bending test.

The results of the study of the mechanical properties of CFRPs are presented in Table 4. The stress–strain curves of the studied systems are shown in Figure 7 to better illustrate the mechanical behavior of CFRPs in the bending test. The strength and bending modulus of the CFRPs practically do not change with an increase in the molar masses of polyimide from sample PI 0.93 to sample PI 0.98 and vary within ≈1560 MPa and ≈117 GPa, respectively (Table 4). However, with a further increase in molecular weight (for the CFRP sample of PI 0.99), a decrease in strength and bending modulus down to 1140 MPa and 101 GPa, respectively, is observed. This decrease is perhaps caused by the deterioration of the impregnation of the carbon fiber by the PI binder, which leads to a reduction in the efficiency of the load transfer from the matrix to the fibers. 

CFRPs obtained on the basis of the low molecular weight homologs for the samples based on PI 0.93 and PI 0.95 have rather low values of interlaminar fracture toughness in the range of 350–370 J/m^2^. As the molecular weight increases, the interlayer viscosity rises significantly to 720 J/g and 830 J/g for PI 0.97 and PI 0.98, respectively. An increase in interlayer fracture toughness is due to a decrease in the brittleness of the polyimide matrix in the higher molecular weight samples. For the CFRP based on high molecular weight polyimide PI 0.99, the highest value of interlaminar fracture toughness of 1800 J/m^2^ is measured. Such a high value of interlayer fracture toughness for the sample of carbon fiber plastic based on PI 0.99 is probably caused by a decrease in brittleness not only because of an increase in the macromolecule length but also due to the absence of the crystalline phase. 

## 4. Conclusions

The samples of linear PAAs were investigated via static and dynamic light scattering and viscometry. The solutions in DMAA were molecularly dispersed. Molar masses of prepared samples were in the range from 22,000 to 70,000 g·mol^−1^. Analysis of molar mass dependences of intrinsic viscosity and hydrodynamic radius showed that the synthesized linear polymers PAAs are typical flexible-chain polymers. The Kuhn segment length is *A* = 2.2 nm.

Based on the PAA of various molar masses, polyimide powders were obtained via chemical cyclization CFRPs were formed from the obtained R-BAPB polyimide powders; for this, a method based on the uniform distribution of polyimide powder on the carbon fibers via electrostatic spraying followed by their impregnation in heated calenders and hot pressing was used. With an increase in the molecular weight, a regular increase in the glass transition and melting temperatures and a decrease in the degree of crystallinity are observed. The lowest degree of crystallinity (1%) is found in the CFRP sample with the highest polyimide molecular weight PI 0.99. CFRPs based on the polyimide sample PI 0.99 have the highest thermal stability, close to 590 °C. Due to the presence of the crystalline phase, the CFRPs based on PI 0.93, PI 0.95, PI 0.97, and PI 0.98 can withstand loads up to temperatures of ≈300 °C. In the case of the CFRP PI 0.99, the operating temperature is limited by its glass temperature. With an increase in the molecular weight of polyimide, an increase in the interlayer fracture toughness of the carbon fiber plastics up to 1800 J/m^2^ is observed. However, the strength and bending modulus somewhat decrease with increasing molecular weight owing to the deterioration of the impregnation of the carbon fibers with the binder because of the high viscosity of the polyimide melt. 

## Figures and Tables

**Table 1:** Designation of the obtained polyamic acids and polyimides.

Molar Ratio of R/BAPB	Name PAA	Name PI
0.93:1	PAA 0.93	PI 0.93
0.95:1	PAA 0.95	PI 0.95
0.97:1	PAA 0.97	PI 0.97
0.98:1	PAA 0.98	PI 0.98
0.99:1	PAA 0.99	PI 0.99

**Table 2:** The molar masses and hydrodynamic characteristics PAAs in DMAA.

Polymers	*M_w_* × 10^–3^, g·mol^−1^	*R*_h-D,_ nm	*f*∙10^−7^ g·c^−1^	*A*_2_∙10^−4^, cm^3^·mole·g^−2^	[η], cm^3^·g^−1^	*N*	*L*, nm
PAA 0.93	22 ± 2	5.1 ± 0.4	0.963	32	45.6 ± 3	30	97
PAA 0.95	26 ± 3	5.7 ± 0.3	1.080	39	56.6 ± 2	35	114
PAA 0.97	29 ± 3	6.1 ± 0.2	1.150	22	58.0 ± 2	40	130
PAA 0.98	36 ± 4	7.0 ± 0.2	1.320	19	72.0 ± 3	49	159
PAA 0.99	70 ± 5	11.0 ± 0.3	2.080	17	110.0 ± 7	95	310

**Table 3:** The thermal properties of the CFRPs based on R-BAPB polymers (Tg is the glass transition temperature, Tm is the melting point, ∆H_m_ is the melting enthalpy of the polymer, χ is the degree of crystallinity, τ5 is the temperature at the loss of 5% of the sample mass).

Sample of CFRP	T_g_, °C	T_m_, °C	∆H_m_, J/g	χ, %	τ_5_, °C
PI 0.93	198	320	42.3	47.0	572
PI 0.95	200	321	40.9	45.4	570
PI 0.97	204	322	37.2	41.3	570
PI 0.98	206	324	36.0	40.0	573
PI 0.99	210	326	1.0	1.1	589

**Table 4:** Mechanical properties of R-BAPB CFRPs with different molecular weights (G_1C_ is the interlaminar fracture toughness, σ_b_ is the bending strength, and E′ is the bending modulus).

Sample of CFRP	G_1c_ J/m^2^	σ_b_, MPa	E′, GPa
PI 0.93	350 ± 25	1590 ± 177	114 ± 7
PI 0.95	370 ± 10	1580 ± 81	118 ± 10
PI 0.97	720 ± 81	1560 ± 81	119 ± 10
PI 0.98	830 ± 13	1510 ± 145	111 ± 6
PI 0.99	1808 ± 65	1146 ± 102	101 ± 5

## Data Availability

Not applicable.

## References

[1] Jin F.-L., Lee S.-Y., Park S.-J., Info A. (2013). Polymer matrices for carbon fiber-reinforced polymer composites. Carbon Lett..

[2] Yao S.S., Jin F.L., Rhee K.Y., Hui D., Park S.J. (2018). Recent advances in carbon-fiber-reinforced thermoplastic composites: A review. Compos. Part B Eng..

[3] Alshammari B.A., Alsuhybani M.S., Almushaikeh A.M., Alotaibi B.M., Alenad A.M., Alqahtani N.B., Alharbi A.G. (2021). Comprehensive Review of the Properties and Modifications of Carbon Fiber-Reinforced Thermoplastic Composites. Polymers.

[4] Vieille B., Taleb L. (2011). About the influence of temperature and matrix ductility on the behavior of carbon woven-ply PPS or epoxy laminates: Notched and unnotched laminates. Compos. Sci. Technol..

[5] Gabrion X., Placet V., Trivaudey F., Boubakar L. (2016). About the thermomechanical behaviour of a carbon fibre reinforced high-temperature thermoplastic composite. Compos. Part B Eng..

[6] Chukov D., Nematulloev S., Zadorozhnyy M., Tcherdyntsev V., Stepashkin A., Zherebtsov D. (2019). Structure, Mechanical and Thermal Properties of Polyphenylene Sulfide and Polysulfone Impregnated Carbon Fiber Composites. Polymers.

[7] Gao S.L., Kim J.K. (2001). Cooling rate influences in carbon fibre/PEEK composites. Part II: Interlaminar fracture toughness. Compos. -Part A Appl. Sci. Manuf..

[8] Bashford D. (1997). Polyphenylene Oxides (PPO). Thermoplastics.

[9] Lu Y.H., Zhan M.S., Zheng W.H. (2006). Preparation and properties of T300 carbon fiber-reinforced thermoplastic polyimide composites. J. Appl. Polym. Sci..

[10] Zhang R., Fallon J.J., Joseph R.M., Thomas J.A., Hassan M.S., Choudhury S.R., Gilmer E.L., Kubota M., Deitzel J.M., Riffle J.S. (2018). Preparation of Submicrometer High-Performance Poly(ether imide) Particles for Fabricating Carbon Fiber Reinforced Polymer Composites. Ind. Eng. Chem. Res..

[11] Ivanov D.A., Matyjaszewski K., Möller M. (2012). Semicrystalline Polymers. Polymer Science: A Comprehensive Reference.

[12] Bessonov M.I., Koton M.M., Kudryavtsev V.V., Laius L.A. (1987). Polyimides–Thermally Stable Polymers.

[13] Andrews E.H. (1974). Morphology and Mechanical Properties in Semicrystalline Polymers. Pure Appl. Chem..

[14] Vaganov G., Didenko A., Ivan’Kova E., Popova E., Yudin V., Elokhovskii V., Lasota I. (2019). Development of new polyimide powder for selective laser sintering. J. Mater. Res..

[15] Vaganov G., Ivan’kova E., Didenko A., Popova E., Elokhovskiy V., Kasatkin I., Yudin V. (2022). High-performance crystallized composite carbon nanoparticles/polyimide fibers. J. Appl. Polym. Sci..

[16] Yudin V., Svetlichnyi V., Gubanova N., Didenko A., Popova E., Sukhanova T., Grigoriev A., Kostereva T., Arbel I., Marom G. (2005). Influence of crystallinity of R-BAPB-type polyimide matrix on thermal and mechanical properties of carbon-fiber-reinforced composites. Polyimides and Other High Temperature Polymers.

[17] Ma Y., Bi Y., Zhang X., Wang D., Dang G., Zhou H., Chen C. (2016). Effects of molecular weight on the dynamic mechanical properties and interfacial properties of carbon fiber fabric-reinforced polyetherketone cardo composites. High Perform. Polym..

[18] Sharma M., Bijwe J. (2012). Influence of molecular weight on performance properties of polyethersulphone and its composites with carbon fabric. Wear.

[19] Tanaka K., Koriyama H., Isshiki S., Katayama T., Shinohara M. (2014). Effect Of The Molecular Weight of Polycarbonate on the Impact Resistance of Continuous CarbonFiber Reinforced Polycarbonate Composites. WIT Trans. Built Environ..

[20] Ye L., Friedrich K., Cutolo D., Savadori A. (1994). Manufacture of CF/PEEK composites from powder/sheath fibre preforms. Compos. Manuf..

[21] Goud V., Alagirusamy R., Das A., Kalyanasundaram D. (2018). Dry Electrostatic Spray Coated Towpregs for Thermoplastic Composites. Fibers Polym..

[22] Vaidya U.K., Chawla K.K. (2008). Processing of fibre reinforced thermoplastic composites. Int. Mater. Rev..

[23] Vaganov G., Yudin V., Vuorinen J., Molchanov E. (2016). Influence of multiwalled carbon nanotubes on the processing behavior of epoxy powder compositions and on the mechanical properties of their fiber reinforced composites. Polym. Compos..

[24] Chu B. (1985). Light Scattering Studies of Polymer Solutions and Melts. Polym. J..

[25] Schärtl W. (2007). Light Scattering from Polymer Solutions and Nanoparticle Dispersions.

[26] Kratochvíl P. (1987). Classical Light Scattering from Polymer Solutions.

[27] Tsvetkov V.N. (1989). Rigid-Chain Polymers: Hydrodynamic and Optical Properties in Solution: T︠S︡vetkov, V.N. (Viktor Nikolaevich): Free Download, Borrow, and Streaming: Internet Archive.

[28] Tsvetkov V.N., Lavrenko P.N., Bushin S.V. (1984). Hydrodynamic invariant of polymer molecules. J. Polym. Sci. Part A.

[29] Tsvetkov V.N., Lavrenko P.N., Bushin S. (1982). V A Hydrodynamic Invariant of Polymeric Molecules. Russ. Chem. Rev..

[30] Simonova M., Kamorin D., Sadikov A., Filippov A., Kazantsev O. (2022). The Influence of Synthesis Method on Characteristics of Buffer and Organic Solutions of Thermo-and pH-Responsive Poly(N-[3-(diethylamino)propyl]methacrylamide)s. Polymers.

[31] Simonova M., Kamorin D., Kazantsev O., Nepomnyashaya M., Filippov A. (2021). Conformation, self-organization and thermoresponsibility of polymethacrylate molecular brushes with oligo(Ethylene glycol)-block-oligo(propylene glycol) side chains. Polymers.

[32] Filippov A.P., Zamyshlyaeva O.G., Tarabukina E.B., Simonova M.A., Kozlov A.V., Semchikov Y.D. (2012). Structural and conformational properties of hyperbranched copolymers based on perfluorinated germanium hydrides1. Polym. Sci.-Ser. A.

[33] Simonova M.A., Tarasova E.V., Dudkina M.M., Tenkovtsev A.V., Filippov A.P. (2019). Synthesis and hydrodynamic and conformation properties of star-shaped polystyrene with calix[8]arene core. Int. J. Polym. Anal. Charact..

[34] Stockmayer W.H., Fixman M. (1963). On the estimation of unperturbed dimensions from intrinsic viscositiesxcin. J. Polym. Sci. Part C Polym. Symp..

[35] Cowie J.M.G., Bywater S. (1965). The use of frictional coefficients to evaluate unperturbed dimensions in dilute polymer solutions. Polymer.

[36] Radu-Dan R., Damaceanu M., Bruma M., Ronova I.A. (2011). Effect of Conformational Parameters on Thermal Properties of Some Poly(oxadiazole-naphthylimide)s. Eur. Polym. J..

[37] Cho H., Kim Y.C., Kim S., Chung I. (2000). Persistence length calculation from light scattering and intrinsic viscosity of dilute semiflexible polyimide solutions with different degree of imidization. Korea-Aust. Rheol. J..

[38] Ronova I.A., Dubronna L.V., Kovalevskii A.Y., Hamciuc C., Bruma M. (1998). The effect of side substituents on rotation hindrance in polyheteroarylenes. Russ. Chem. Bull..

[39] Tarabukina E., Amirova A., Belyaeva E., Krasova A., Simonova M., Filippov A., Meleshko T., Ilgach D., Bogorad N., Yakimansky A. (2013). Conformational Characteristics of Polyimide Initiator for the Synthesis of Poly(Methylmethacrylate) Grafted Block-Copolymers. J. Macromol. Sci. Part B.

[40] Birshtein T.M., Goriunov A.M. (1979). The theoretical analysis of the elasticity of polyimides and polyaminoacids. Polym. Sci. Ser. A.

[41] Lyulin S.V., Larin S.V., Gurtovenko A.A., Lukasheva N.V., Yudin V.E., Svetlichnyi V.M., Lyulin A.V. (2012). Effect of the SO_2_ group in the diamine fragment of polyimides on their structural, thermophysical, and mechanical properties. Polym. Sci.-Ser. A.

[42] Van Krevelen D.W., Te Nijenhuis K. (2009). Rheological Properties of Polymer Melts. Properties of Polymers.

[43] Botelho E.C., Figiel Ł., Rezende M.C., Lauke B. (2003). Mechanical behavior of carbon fiber reinforced polyamide composites. Compos. Sci. Technol..

[44] Durai Prabhakaran R.T., Pillai S., Charca S., Oshkovr S.A., Knudsen H., Andersen T.L., Ilsted Bech J., Thomsen O.T., Lilholt H. (2015). Mechanical characterization and fractography of glass fiber/polyamide (PA6) composites. Polym. Compos..

